# Association Between Sex-Specific Risk Factors and Risk of New-Onset Atrial Fibrillation Among Women

**DOI:** 10.1001/jamanetworkopen.2022.29716

**Published:** 2022-09-01

**Authors:** Zuolin Lu, Elif Aribas, Sven Geurts, Jeanine E. Roeters van Lennep, M. Arfan Ikram, Maxime M. Bos, Natasja M. S. de Groot, Maryam Kavousi

**Affiliations:** 1 Department of Epidemiology, Erasmus MC, University Medical Center Rotterdam, Rotterdam, the Netherlands; 2 Department of Internal Medicine, Erasmus MC, University Medical Center Rotterdam, Rotterdam, the Netherlands; 3 Department of Cardiology, Erasmus MC, University Medical Center Rotterdam, Rotterdam, the Netherlands

## Abstract

**Importance:**

Atrial fibrillation (AF) is the most common cardiac arrhythmia worldwide, with different epidemiological and pathophysiological processes for women vs men and a poorer prognosis for women. Further investigation of sex-specific risk factors associated with AF development in women is warranted.

**Objective:**

To investigate the linear and potential nonlinear associations between sex-specific risk factors and the risk of new-onset AF in women.

**Design, Setting, and Participants:**

This population-based cohort study obtained data from the 2006 to 2010 UK Biobank study, a cohort of more than 500 000 participants aged 40 to 69 years. Participants were women without AF and history of hysterectomy and/or bilateral oophorectomy at baseline. Median follow-up period for AF onset was 11.6 years, and follow-up ended on October 3, 2020.

**Exposures:**

Self-reported, sex-specific risk factors, including age at menarche, history of irregular menstrual cycle, menopause status, age at menopause, years after menopause, age at first live birth, years after last birth, history of spontaneous miscarriages, history of stillbirths, number of live births, and total reproductive years.

**Main Outcomes and Measures:**

The primary outcome was new-onset AF, which was defined by the use of *International Statistical Classification of Diseases and Related Health Problems, Tenth Revision* code I48.

**Results:**

A total of 235 191 women (mean [SD] age, 55.7 [8.1] years) were included in the present study. During follow-up, 4629 (2.0%) women experienced new-onset AF. In multivariable-adjusted models, history of irregular menstrual cycle was associated with higher AF risk (hazard ratio [HR], 1.34; 95% CI, 1.01-1.79). Both early menarche (age 7-11 years; HR, 1.10 [95% CI, 1.00-1.21]) and late menarche (age 13-18 years; HR, 1.08 [95% CI, 1.00-1.17]) were associated with AF incidence. Early menopause (age 35-44 years; HR, 1.24 [95% CI, 1.10-1.39]) and delayed menopause (age ≥60 years; HR, 1.34 [95% CI, 1.10-1.78]) were associated with higher risk of AF. Compared with women with 1 to 2 live births, those with 0 live births (HR, 1.13; 95% CI, 1.04-1.24) or 7 or more live births (HR, 1.67; 95% CI, 1.03-2.70) both had significantly higher AF risk.

**Conclusions and Relevance:**

Results of this study suggest that irregular menstrual cycles, nulliparity, and multiparity were associated with higher risk of new-onset AF among women. The results highlight the importance of taking into account the reproductive history of women in devising screening strategies for AF prevention.

## Key Points

**Question:**

What are the linear and nonlinear associations between various sex-specific risk factors and atrial fibrillation (AF) onset in women?

**Findings:**

In this cohort study of 235 191 women without AF at baseline, history of early or delayed menopause or irregular menstrual cycles was significantly associated with higher risk of new-onset AF. Both nulliparity and multiparity were also significantly associated with a higher AF risk.

**Meaning:**

Findings of this study suggest that AF screening and prevention strategies need to take into account the reproductive history of women.

## Teaser

This cohort study identifies the reproductive variables associated with atrial fibrillation development among women aged 40 to 69 years.

## Introduction

Atrial fibrillation (AF) is the most common cardiac arrhythmia worldwide and carries a large morbidity and mortality risk.^1^ Evidence suggests differences in the pathophysiological processes of AF between men and women and an association of AF with a poor prognosis among women.^1,2^ Such findings warrant additional research into the sex-specific risk factors in the development of AF.

Sex hormones may play a key role in cardiovascular health.^3^ The suggested benefits of estrogen for cholesterol metabolism and endothelial function^4^ diminish as women age. This age-related decline in estrogen levels, particularly after menopause, has been associated with a higher risk of cardiovascular disease (CVD).^5^

The pathophysiological processes of AF are known to be complex and multifaceted. An electrophysiological dysfunction within the heart, including a disordered refractory period and action potential duration, is thought to be one of the most important factors in initiating AF.^2^ Despite the lack of direct evidence, estrogen may confer an advantage in AF by extending atrial conduction time, action potential duration, and the atrial effective refractory period.^6^ Thus, we speculated that reproductive life span function is potentially associated with AF development in women, induced by the long-lasting changes in estrogen levels related to aging.

Although associations of menopausal age and reproductive life span with incident AF have been reported,^7,8,9^ a comprehensive evaluation of the potential association of a wide range of reproductive life span factors with AF development is sparse. In the present study, we aimed to investigate the linear and potential nonlinear associations between sex-specific risk factors and the risk of new-onset AF among a large population of women in the UK Biobank study.

## Methods

### Study Population

Data were obtained from the UK Biobank database. The UK Biobank is a large, prospective population-based cohort study in the UK that recruited more than 500 000 participants aged 40 to 69 years in 2006 to 2010.^10^ These participants provided medical history, health behavior, physical measures, and biological samples at the time of enrollment. The UK Biobank received ethics approval from the North West Multi-Centre Research Ethics Committee, the National Information Governance Board for Health and Social Care in England and Wales, and the Community Health Index Advisory Group in Scotland. All participants provided written informed consent before inclusion in the UK Biobank, and any participant who withdrew from the study was removed from the present cohort study. We followed the Strengthening the Reporting of Observational Studies in Epidemiology (STROBE) reporting guideline.

**Figure 1.**
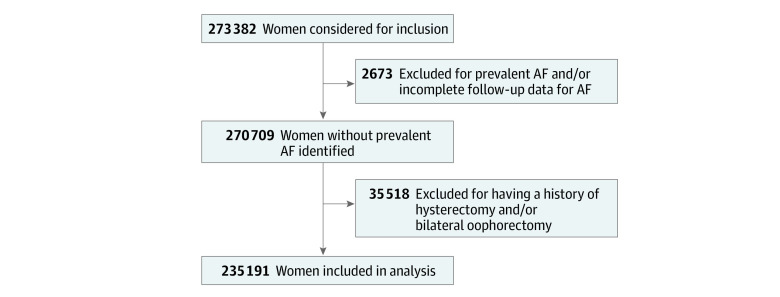
Flowchart of the Study Population AF indicates atrial fibrillation.

In the current study, 273 382 female participants at study enrollment were assessed for inclusion. Participants with prevalent AF at baseline or with only self-reported incident AF during follow-up were excluded (n = 2673). Furthermore, participants with a history of hysterectomy and/or bilateral oophorectomy were excluded (n = 35 518). The number of participants in each analysis for the various sex-specific risk factors varied because of missing values per specific risk factor (Figure 1).

### Assessment of Sex-Specific Risk Factors, AF, and Cardiovascular Risk Factors

Reproductive history was self-reported by participants at the baseline study visit.^9^ Potential sex-specific risk factors included in the current study were age at menarche, history of irregular menstrual cycle (yes or no), menopause status (yes or no), age at menopause, years after menopause (calculated as baseline age minus menopausal age), age at first live birth, years after last birth (calculated as baseline age minus age at last birth), history of spontaneous miscarriages (yes or no), history of stillbirths (yes or no), number of live births, and total reproductive years (calculated as menopausal age minus menarcheal age).^11,12^

The primary outcome was new-onset AF. Atrial fibrillation was assessed using the hospital admission, primary care, and/or death registry data linked to the UK Biobank.^10^ Onset of AF was defined by the use of *International Statistical Classification of Diseases and Related Health Problems, Tenth Revision* code I48. Follow-up ended on October 3, 2020. Participants were censored at the end of follow-up, date of incident AF, date of death, or loss to follow-up, whichever occurred first.

Assessment of potential confounders at baseline has been described previously.^10^ The details are provided in the eMethods in the Supplement.

### Statistical Analysis

Multivariable Cox proportional hazards regression models were used to quantify associations between each risk factor and incident AF. All risk factors were first treated as continuous variables in the Cox proportional hazards regression model. In model 1, we adjusted the analyses for baseline age only. In model 2, we also adjusted for cardiovascular risk factors such as race and ethnicity (which were self-identified by participants and included Asian, Black, White, mixed, and other [ie, all other potential racial and ethnic groups]), educational level, body mass index (BMI), total cholesterol, high-density lipoprotein cholesterol, systolic blood pressure, diastolic blood pressure, smoking status, history of diabetes, history of coronary heart disease (CHD), history of heart failure, history of stroke, use of blood pressure–lowering medication, use of cholesterol-lowering medication, use of hormone replacement therapy (if applicable), and use of contraceptive medication (if applicable).

In sensitivity analyses, we repeated all of the analyses among participants without CVD (including CHD, heart failure, and stroke) at baseline to ascertain the presence of any residual confounding despite the extensive adjustments. Furthermore, we stratified analyses by BMI (calculated as weight in kilograms divided by height in meters squared) categories: (1) underweight: BMI lower than 18.5; (2) healthy weight: BMI between 18.5 and lower than 25; (3) overweight: BMI between 25 and lower than 30; (4) obese: BMI of 30 or higher. To investigate the role of sex hormones in the linear association between sex-specific risk factors and AF, we also adjusted model 2 for the serum concentrations of testosterone and sex hormone–binding globulin instead of estradiol because estradiol was present in a small proportion of the participants (6.2% [14 588]) in the UK Biobank. Moreover, given that women without live birth may have infertility induced by hormonal imbalance or may have had pregnancy loss, we performed a sensitivity analysis by further adjusting for sex hormone levels and a subgroup analysis among women without stillbirth, spontaneous miscarriage, or termination. In addition, we recognized that several of the assessed risk factors inherently captured the aging process. For instance, postmenopausal women are expected to be older than premenopausal women. Therefore, age-stratified analysis with 5-year age groups was conducted to limit the residual confounding of age.

Furthermore, we added natural cubic splines with up to 5 knots to the corresponding multivariable-adjusted Cox proportional hazards regression models for each risk factor to ascertain the potential nonlinear associations of factors with incident AF. The Akaike information criterion, an estimator of how well a model fits the data, was used to compare the various models and choose the best model. We then recorded the cutoff value of each risk factor if nonlinearity was found. The cutoff value was used to group participants and construct the categorical variables, which were subsequently used in the Cox proportional hazards regression models to quantify the nonlinear associations.

Missing covariate values were imputed under the assumption of missing at random using the multiple imputation with fully conditional specification in the R package mice (R Foundation for Statistical Computing). High-density lipoprotein cholesterol levels were missing in 15.3% of participants. Missing values of all other covariables were 8.0% or less. For multiple imputation, all available data were used to generate 5 imputed data sets, and the pooled results were reported. In sensitivity analyses, a complete case analysis was carried out.

Statistical significance was considered to be 2-tailed *P* < .05. The analyses were performed with R, version 4.0.2 (R Foundation for Statistical Computing).

## Results

A total of 235 191 women without AF (mean [SD] age, 55.7 [8.1] years) at baseline were included in the study. The self-reported baseline characteristics of participants are shown in Table 1. The median (IQR) follow-up period was 11.6 (10.9-12.3) years, during which 4629 (2.0%) women experienced new-onset AF.

**Table 1.** Baseline Characteristics of the Study Population

Characteristic	No. (%)
No. of participants	235 191 (100)
Age, mean (SD), y	55.7 (8.1)
Weight, mean (SD), kg	71.1 (14.0)
BMI, mean (SD)	26.9 (5.1)
Blood pressure, mean (SD), mm Hg	
Systolic	136.5 (20.2)
Diastolic	80.5 (10.5)
Total cholesterol, mean (SD), mg/dL	226.3 (43.2)
HDL cholesterol, mean (SD), mg/dL	61.8 (14.7)
Total estradiol, median (IQR), pg/mL	109.7 (73.1-175.4)
Total testosterone, median (IQR), ng/dL	29.7 (21.0-40.1)
SHBG, mean (SD), μg/mL	6.97 (3.42)
Race and ethnicity^a^	
Asian	3964 (1.7)
Black	3905 (1.7)
White	222 577 (94.6)
Mixed	1650 (0.7)
Other^b^	3095 (1.3)
University or college educational level	36 938 (15.7)
Smoking status	
Never	141 240 (60.1)
Former	72 888 (31.0)
Current	21 063 (9.0)
Disease history	
Diabetes	7549 (3.2)
Heart failure	384 (0.2)
CHD	5916 (2.5)
Stroke	2430 (1.0)
Medication use	
Blood pressure lowering	44 798 (19.0)
Cholesterol lowering	51 795 (22.0)
Oral contraceptive	51 238 (21.8)
Hormone replacement therapy	76 438 (32.6)
Age at menarche, mean (SD), y	12.9 (1.6)
History of irregular menstrual cycle	10 804 (22.5)
With menopause status	143 067 (69.2)
Age at menopause, mean (SD), y	50.2 (4.4)
Years after menopause, mean (SD), No.	9.6 (6.3)
Age at first live birth, mean (SD), y	25.6 (4.7)
History of spontaneous miscarriages	46 972 (23.5)
History of stillbirths	5655 (2.4)
No. of live births	
0	45 641 (19.5)
1	31 838 (13.6)
2	102 185 (43.6)
≥3	54 867 (23.3)
Total reproductive years, mean (SD)	37.3 (4.7)

Abbreviations: BMI, body mass index (calculated as weight in kilograms divided by height in meters squared); CHD, coronary heart disease; HDL, high-density lipoprotein; SHBG, sex hormone–binding globulin.

SI conversion factors: To convert total and HDL cholesterol to millimoles per liter, multiply by 0.0259; estradiol to picomoles per liter, multiply by 3.671; testosterone to nanomoles per liter, multiply by 0.0347; and SHBG to nanomoles per liter, multiply by 8.896.

^a^ Race and ethnicity were self-identified by participants.

^b^ Other category included all other potential racial and ethnic groups.

### Linear Associations 

Table 2 describes associations between various sex-specific risk factors and the risk of new-onset AF among participants. In the age-adjusted model (model 1), associations were found between most assessed risk factors and incident AF with the exception of menopause status (hazard ratio [HR], 1.04; 95% CI, 0.90-1.20), history of spontaneous miscarriage (HR, 1.05; 95% CI, 0.98-1.13), and history of stillbirth (HR, 1.17; 95% CI, 1.00-1.38). After additional adjustments for other potential confounders (model 2), the associations between age at menarche and number of live births with AF were attenuated and were no longer statistically significant.

**Table 2.** Linear Association Between Sex-Specific Risk Factors and Risk of New-Onset Atrial Fibrillation

Characteristic	No. of participants with data	HR (95% CI)			
		Model 1^a^	*P* value	Model 2^b^	*P* value
Age at menarche^c^	227 319	0.98 (0.96-0.99)	.01	1.00 (0.98-1.02)	.99
History of irregular menstrual cycle, yes or no	58 843	1.36 (1.02-1.81)	.04	1.34 (1.01-1.79)	.04
Menopause status, yes or no	206 886	1.04 (0.90-1.20)	.63	1.14 (0.98-1.32)	.09
Age at menopause^d^	134 419	0.94 (0.90-0.97)	<.001	0.95 (0.92-0.98)	<.01
Years after menopause^d^^,^^e^	134 419	1.07 (1.03-1.11)	<.001	1.05 (1.02-1.09)	<.01
Age at first live birth^d^	156 773	0.86 (0.82-0.89)	<.001	0.92 (0.88-0.96)	<.001
Years after last birth^d^^,^^f^	156 527	1.09 (1.05-1.13)	<.001	1.06 (1.02-1.10)	<.01
History of spontaneous miscarriages, yes or no	230 587	1.05 (0.98-1.13)	.20	1.04 (0.97-1.11)	.32
History of stillbirths, yes or no	230 953	1.17 (1.00-1.38)	.05	1.07 (0.91-1.26)	.39
No. of live births	234 531	1.03 (1.01-1.05)	.03	1.01 (0.98-1.03)	.57
Total reproductive years^d^^,^^g^	131 449	0.96 (0.93-0.99)	.02	0.96 (0.93-0.99)	.02

Abbreviation: HR, hazard ratio.

^a^ Model 1 was adjusted for baseline age only.

^b^ Model 2 was adjusted for baseline age, race and ethnicity, educational level, body mass index, total cholesterol, high-density lipoprotein cholesterol, systolic blood pressure, diastolic blood pressure, smoking status, history of diabetes, history of coronary heart disease, history of heart failure, history of stroke, use of blood pressure–lowering medication, use of cholesterol-lowering medication, use of hormone therapy (if applicable), and use of contraceptive medication (if applicable).

^c^ HRs represent a 1-unit increase in association of age at menarche with the risk of new-onset atrial fibrillation.

^d^ HRs represent a 5-unit increase in association of age at menopause, years after menopause, age at first live birth, and reproductive years with the risk of new-onset atrial fibrillation.

^e^ Calculated as baseline age minus menopausal age.

^f^ Calculated as baseline age minus age at last birth.

^g^ Defined as the difference between menopausal age and menarcheal age.

In model 2, women with a history of irregular menstrual cycles had a higher risk of incident AF compared with women with regular menstrual cycles (HR, 1.34; 95% CI, 1.01-1.79). A greater number of years after last birth was associated with higher AF risk (HR, 1.06 [95% CI, 1.02-1.10] per 5-year increase in years after last birth). Older age at menopause was beneficial for incident AF (HR, 0.95 [95% CI, 0.92-0.98] per 5-year increase in menopausal age), whereas a greater number of years after menopause was detrimental to the association with new-onset AF (HR, 1.05 [95% CI, 1.02-1.09] per 5-year increase in years after menopause). Older age at first live birth was associated with a lower risk of incident AF (HR, 0.92 [95% CI, 0.88-0.96] per 5-year increase in age at first live birth). Moreover, longer reproductive life span, reflecting the period between menarche and menopause, was associated with lower risk of incident AF (HR, 0.96 [95% CI, 0.93-0.99] per 5-year increase in total reproductive years).

### Nonlinear Associations Between Sex-Specific Risk Factors and Risk of New-Onset AF

**Figure 2.**
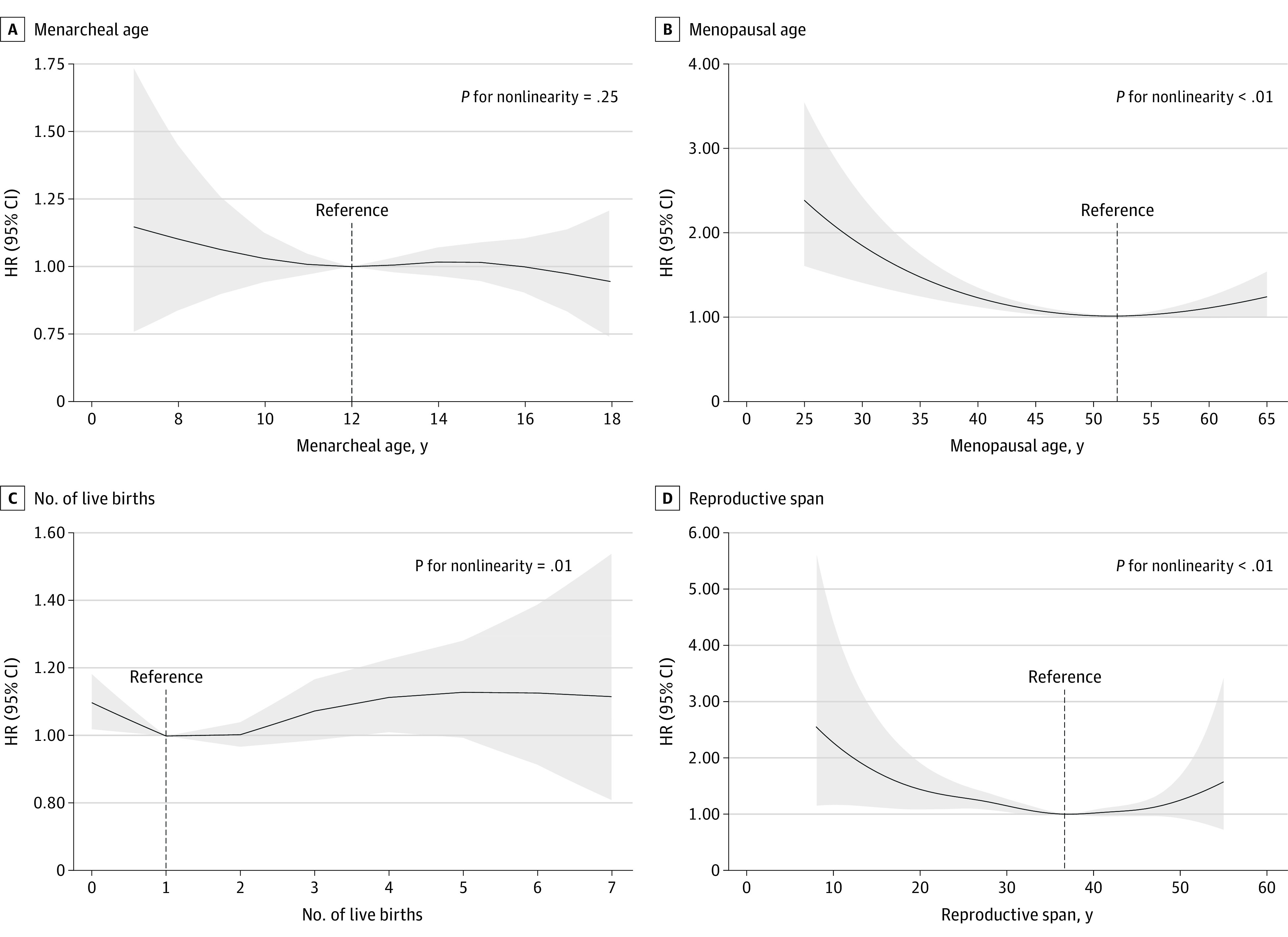
Nonlinear Association Between Sex-Specific Risk Factors and Risk of New-Onset Atrial Fibrillation Model was adjusted for baseline age, race and ethnicity, educational level, body mass index, total cholesterol, high-density lipoprotein cholesterol, systolic blood pressure, diastolic blood pressure, smoking status, history of diabetes, history of coronary heart disease, history of heart failure, history of stroke, use of blood pressure–lowering medication, and use of cholesterol-lowering medication. Shaded areas indicate 95% CIs. HR indicates hazard ratio.

Figure 2 depicts the nonlinear association between sex-specific risk factors and AF development among participants. An N-shaped association was found between age at menarche and incident AF (*P* for nonlinearity = .25). Experiencing menarche earlier between ages 7 and 11 years (HR, 1.10; 95% CI, 1.00-1.21) or later between ages 13 and 18 years (HR, 1.08; 95% CI, 1.00-1.17) was associated with a higher risk of incident AF compared with menarche at age 12 years (Table 3, Figure 2A). Moreover, a U-shaped association was identified between menopausal age and the risk of new-onset AF. Experiencing menopause at approximately 52 years of age was associated with the lowest risk for incident AF (Figure 2B). Early menopause (age <35 years: HR, 2.25 [95% CI, 1.48-3.43]; age 35-44 years: HR, 1.24 [95% CI, 1.10-1.39]) and menopause at 60 years or older (HR, 1.34; 95% CI, 1.10-1.78) were associated with a higher risk for incident AF (Table 3).

**Table 3.** Nonlinear Association Between Sex-Specific Risk Factors With Risk of New-Onset Atrial Fibrillation

Characteristic	No. of participants	HR (95% CI)			
		Model 1^a^	*P* value	Model 2^b^	*P* value
Age at menarche, y					
7-11	44 309	1.18 (1.07-1.29)	<.001	1.10 (1.00-1.21)	.04
12	43 314	1 [Reference]	NA	1 [Reference]	NA
13-18	139 696	1.04 (0.96-1.13)	.34	1.08 (1.00-1.17)	.05
Age at menopause, y					
<35	494	2.48 (1.63-3.78)	<.001	2.25 (1.48-3.43)	<.001
35-44	12 074	1.33 (1.19-1.49)	<.001	1.24 (1.10-1.39)	<.001
45-49	32 042	1.09 (1.00-1.83)	.06	1.07 (0.98-1.17)	.12
50-54	68 206	1 [Reference]	NA	1 [Reference]	NA
55-59	20 665	1.06 (0.97-1.17)	.20	1.04 (0.95-1.14)	.39
≥60	938	1.39 (1.05-1.84)	.02	1.34 (1.01-1.78)	.04
No. of live births					
0	45 641	1.09 (1.00-1.19)	.05	1.13 (1.04-1.24)	<.01
1-2	134 023	1 [Reference]	NA	1 [Reference]	NA
3	40 695	1.11 (1.02-1.20)	.01	1.08 (1.00-1.16)	.05
4-6	13 784	1.23 (1.10-1.37)	<.001	1.12 (1.01-1.24)	.04
≥7	388	1.87 (1.18-2.98)	<.001	1.67 (1.03-2.70)	.03
Total reproductive years^c^					
≤20	462	1.87 (1.14-3.06)	.01	1.74 (1.07-2.86)	.03
21-30	10 863	1.31 (1.16-1.47)	<.001	1.23 (1.10-1.38)	<.001
31-40	88 122	1 [Reference]	NA	1 [Reference]	NA
41-50	31 939	1.07 (0.99-1.16)	.09	1.03 (0.95-1.11)	.45

Abbreviations: HR, hazard ratio; NA, not applicable.

^a^ Model 1 was adjusted for baseline age only.

^b^ Model 2 was also adjusted for race and ethnicity, educational level, body mass index, total cholesterol, high-density lipoprotein cholesterol, systolic blood pressure, diastolic blood pressure, smoking status, history of diabetes, history of coronary heart disease, history of heart failure, history of stroke, use of blood pressure–lowering medication, and use of cholesterol-lowering medication.

^c^ Defined as the difference between menopausal age and menarcheal age.

Figure 2C illustrates a J-shaped association between number of live births and incident AF. The lowest risk of AF was observed among women with 1 to 2 live births. Compared with women who had 1 or 2 live births, those with none had a higher risk of incident AF (HR, 1.13; 95% CI, 1.04-1.24). Risk of AF was also higher among women with 4 to 6 live births (HR, 1.12; 95% CI, 1.01-1.24) and substantially higher among women with 7 or more live births (HR, 1.67; 95% CI, 1.03-2.70).

We also observed a U-shaped association between reproductive life span and the risk of new-onset AF (Figure 2D). In Table 3, short reproductive life spans of 20 years or less (HR, 1.74; 95% CI, 1.07-2.86) and 21 to 30 years (HR, 1.23; 95% CI, 1.10-1.38) were markedly associated with higher AF risks. In contrast, a reproductive life span of 41 years or longer was not associated with AF.

### Sensitivity Analyses

The complete case analysis showed generally similar directions compared with the results after multiple imputation. However, no association was found between menarcheal age and AF (eTable 1 in the Supplement). After excluding women with baseline prevalent CHD, heart failure, and stroke, the associations between various sex-specific risk factors and incident AF remained significant and were similar to the original results (eTable 2 in the Supplement). For example, history of irregular menstrual cycle was significantly associated with higher AF risk (HR, 1.34; 95% CI, 1.01-1.79). Moreover, we found a significant interaction (*P* for interaction <.001) between menopause status and incident AF across BMI categories (eTable 3 in the Supplement). Menopause status was associated with incident AF only among those in the healthy weight BMI group (HR, 1.39; 95% CI, 1.05-1.84). Further adjustment for blood levels of sex hormones did not substantially change the associations between each risk factor and AF (eTable 4 in the Supplement). Specifically, compared with women with 1 to 2 live births, nulliparity was associated with a higher AF risk (HR, 1.10; 95% CI, 1.01-1.19; *P* = .03).

Age-stratified analyses suggested significant interactions between history of irregular menstrual cycle and baseline age (age ≤45 years: HR, 0.76 [95% CI, 0.36-1.61]; age 46-50 years: HR, 1.30 [95% CI, 0.83-2.03]; and age 51-55 years: HR, 2.37 [95% CI, 1.40-4.02]; *P* for interaction = .03). In addition, a potential association was found between menopausal age and AF among younger women, although the interaction was not statistically significant. Borderline significant interactions were also observed between age and menopause status (age ≤45 years: HR, 2.95 [95% CI, 1.26-6.89]; age 46-50 years: HR, 1.18 [95% CI, 0.79-1.77]; age 51-55 years: HR, 1.35 [95% CI, 1.00-1.82]; age 56-60 years: HR, 1.21 [95% CI, 0.62-2.34]; age 61-65 years: HR, 0.79 [95% CI, 0.47-1.31]; and age >65 years: HR, 0.96 [95% CI, 0.64-1.45]; *P* for interaction = .09) and years after menopause (age 46-50 years: HR, 1.51 [95% CI, 1.08-2.11]; age 51-55 years: HR, 1.14 [95% CI, 0.98-1.34]; age 56-60 years: HR, 1.13 [95% CI, 1.04-1.23]; age 61-65 years: HR, 1.00 [95% CI, 0.94-1.06]; and age >65 years: HR, 1.05 [95% CI, 0.99-1.11]; *P* for interaction = .18) (eTable 5 in the Supplement).

To evaluate the additional value of the reproductive life span beyond menopausal age, we adjusted for menopausal age in the association between total reproductive years and new-onset AF; this association remained, although it was slightly attenuated (age 21-30 years: HR, 1.19; 95% CI, 1.01-1.40; *P* = .04) (eTable 6 in the Supplement). After excluding women with a history of pregnancy loss, an association was found between number of live births and AF (0 live births: HR, 1.15 [95% CI, 1.06-1.27]; 3 live births: HR, 1.14 [95% CI, 1.04-1.25]; and 4-6 live births: HR, 1.16 [95% CI, 1.01-1.34]) (eTable 7 in the Supplement). Further adjustment for the Townsend index did not change the significance. Compared with women who had 1 or 2 live births, those with 0 live births had a higher risk of incident AF (HR, 1.09; 95% CI, 1.00-1.18). Risk of AF was also higher among women with 3 live births (HR, 1.08; 95% CI, 1.00-1.16) and among women with 4 to 6 live births (HR, 1.13; 95% CI, 1.01-1.26) (eTable 7 in the Supplement).

## Discussion

This study found independent significant linear associations between risk of new-onset AF and the risk factors of age at menopause, years after menopause, total reproductive years, history of irregular menstrual cycle, number of live births, age at first live birth, and years after last birth. Significant nonlinear associations were also found between risk of new-onset AF and age at menopause, total reproductive years, and number of live births.

To our knowledge, this study was the first to report an independent association between irregularity in menstrual cycle and new-onset AF and thereby add to previous evidence. Epidemiological studies have reported that irregular cycles might be associated with the development of CHD and CHD mortality.^13,14^ Meanwhile, the findings of a study of 40 Indian women suggested that menstrual cycle irregularity was associated with glucose intolerance and insulin resistance.^15^ Irregular cycles that are commonly induced by sex-hormone disorders were considered to be an independent risk factor for cardiometabolic disorders.^16^ In addition, the observed association in the present study was significant only among older women, suggesting that loss of estrogen with aging might mediate the association between history of irregular menstrual cycle and new-onset AF.^5,6^ However, further research to investigate the underlying pathophysiological mechanisms between irregular cycles and AF are warranted.

Although menopause status was not associated with new-onset AF in the present study, a greater number of years after menopause was associated with a higher AF risk among postmenopausal women. Moreover, the risk of AF was significantly increased among women who had experienced menopause at 44 years or younger or at 60 years or older. These results reflected a U-shaped association, with the lowest AF-associated risk found for the menopausal ages of 45 to 59 years, and thus were complementary to results of a previous study conducted within the UK Biobank that showed linear associations between menopausal age and incident AF among women with natural menopause or surgical menopause.^9^ However, analyses of the Framingham Heart Study of 1809 women (median follow-up of 20.5 years) and the Women’s Health Study (WHS) of 30 034 women (median follow-up of 10 years) did not report an association between categorical menopausal age and new-onset AF.^17,18^ Participants in the Framingham Heart Study (mean age, 70 years) were much older than those in the current study. Advancing age was the most important risk factor for incident AF, leading to a sharp increase in AF incidence after 70 years of age.^1^ Thus, the association between menopausal age and AF could have been masked by both the limited sample size and the older population in the Framingham Heart Study. In the present study, results of the sensitivity analyses suggested an association between menopausal age and AF among younger women. Moreover, the reference group in the WHS was arbitrarily set to women with menopausal age older than 54 years. In this study, we found that menopausal age of 60 years or older was associated with a substantially higher risk of AF. Therefore, not taking into account the nonlinearity in the WHS was probably a factor in the diluted association between menopausal age and AF. We extended the previous studies by assessing the potential nonlinear associations and identifying an appropriate reference group with the lowest risk for AF.

We found a J-shaped association between the number of live births and incident AF. Compared with women with 1 to 2 live births, those with 0 or with more than 4 live births had a higher risk of AF development. To our knowledge, only 1 study from the WHS found that women with more than 3 pregnancies vs 1 pregnancy had a substantially higher AF risk, whereas women without pregnancy did not have a higher AF risk.^19^ Population heterogeneity should be assessed to interpret such a discrepancy. The participants in the WHS were healthier than those in the UK Biobank. Thus, in the present study, the higher risk of AF among women who had 0 live births might be partially attributed to a poorer health status and a larger burden of comorbidities. Nevertheless, the sensitivity analyses of women without CVD at baseline showed similar results, suggesting that the burden of comorbidity did not fully account for the observed association. Compared with women without children, primiparous women experience a series of changes in vascular function during pregnancy, and these changes are normally beneficial to accommodate maternal and fetal needs.^20^ On one hand, research has indicated that a normal pregnancy might contribute to reduced arterial stiffness and elevated vascular compliance in primiparas.^21^ On the other hand, pregnancy may be associated with abnormal hemodynamic changes in the cardiovascular system, which further result in cardiac hypertrophy, valvular disease, and CHD.^22^ Evidence shows that at least 0.2% to 0.4% of all pregnancies are complicated by these cardiovascular pathologies.^23^ Herein, multiparity was associated with increased risk of pathological changes within the cardiovascular system after pregnancy. Overall, the findings of this study underscored the higher AF risk among both nulliparous and multiparous women.

In addition, the present study found associations between various factors of reproductive history and incident AF. Associations of age at first live birth and years after last birth with incident AF were other novel findings of this study. Evidence has shown that women with an early first pregnancy are at greater risk of poor general health and worse physical functioning.^24^ Similar to a prospective Korean study, this study confirmed the inverse association between the number of years in the reproductive life span and incident AF.^8^

### Strengths and Limitations

This study has several strengths. Among these strengths are the large sample size, prospective study design, long-term follow-up, and extensive adjustment for a broad range of confounders.

This study also has several limitations. First, given that participants were predominantly of European ancestry, the results may not be generalizable to women of other ethnic backgrounds. Second, various exposures and covariates were self-reported and thus may be subject to recall bias, which is inherent to the use of self-administered questionnaires. Third, because of the observational study design, we cannot rule out the possibility of residual or unmeasured confounding. Specifically, the association between the number of live births and cardiovascular health might be, to some extent, altered by possible residual confounding by socioeconomic and cultural factors.^25^ However, the observed association remained statistically significant after further adjustment for the Townsend index. Fourth, previous studies^26^ have suggested an increased risk of CVD among women who had experienced adverse pregnancy outcomes such as preeclampsia, preterm delivery, and gestational diabetes, which were not covered in the present study because of the low prevalence of adverse pregnancy outcomes at baseline. Fifth, given that AF may be paroxysmal and asymptomatic, we might have underestimated the true number of AF cases in this study population. Furthermore, the use of *International Statistical Classification of Diseases and Related Health Problems, Tenth Revision* code I48 to detect the onset of AF was tied to health care utilization and not perfectly accurate and was thus at a potential risk of misclassification.^27^

## Conclusions

This large, population-based cohort study found that sex-specific risk factors were associated with the risk of new-onset AF among women. The AF risk was elevated among women with early or delayed menopause. In addition, women with irregular menstrual cycles had a greater risk for AF onset. Both nulliparity and multiparity were associated with greater risk of incident AF compared with having 1 to 2 live births. These results underscored the importance of taking into account the reproductive history of women when developing tailored screening strategies for AF prevention in women.

## Notes

Supplement.
eMethods
**eTable 1.** Complete Case Analysis **eTable 2.** Association Between Sex-Specific Risk Factors With the Risk of New-Onset Atrial Fibrillation Among Participants Free of Cardiovascular Disease at Baseline **eTable 3.** Association Between Various Sex-Specific Risk Factors With the Risk of New-Onset Atrial Fibrillation Stratified by Body Mass Index **eTable 4.** Association Between Sex-Specific Risk Factors With the Risk of New-Onset Atrial Fibrillation Additionally Adjusted for Sex-Hormones **eTable 5.** Association Between Sex-Specific Risk Factors With the Risk of New-Onset Atrial Fibrillation Stratified by Baseline Age Categories **eTable 6.** Sensitivity Analysis: Association Between Categorical Reproductive Span and New-Onset AF After Further Adjustment for Menopausal Age **eTable 7.** Sensitivity Analysis: Association Between Categorical Number of Live Births and New-Onset AF
