# Supplementary material for: Association Between Sex-Specific Risk Factors and Risk of New-Onset Atrial Fibrillation Among Women
Source: JAMA Netw Open. 2022 Sep 1;5(9):e2229716. doi: 10.1001/jamanetworkopen.2022.29716 (PMC9437751; doi:10.1001/jamanetworkopen.2022.29716)
Supplement: Supplement. — eMethods eTable 1. Complete Case Analysis eTable 2. Association Between Sex-Specific Risk Factors With the Risk of New-Onset Atrial Fibrillation Among Participants Free of Cardiovascular Disease at Baseline eTable 3. Association Between Various Sex-Specific Risk Factors With the Risk of New-Onset Atrial Fibrillation Stratified by Body Mass Index eTable 4. Association Between Sex-Specific Risk Factors With the Risk of New-Onset Atrial Fibrillation Additionally Adjusted for Sex-Hormones eTable 5. Association Between Sex-Specific Risk Factors With the Risk of New-Onset Atrial Fibrillation Stratified by Baseline Age Categories eTable 6. Sensitivity Analysis: Association Between Categorical Reproductive Span and New-Onset AF After Further Adjustment for Menopausal Age eTable 7. Sensitivity Analysis: Association Between Categorical Number of Live Births and New-Onset AF [file jamanetwopen-e2229716-s001.pdf]

Supplemental Online Content

Lu Z, Aribas E, Geurts S, et al. Association between sex-specific risk factors and risk of new-onset atrial fibrillation among women. *JAMA Netw Open*. 2022;5(9):e2229716. doi:10.1001/jamanetworkopen.2022.29716

eMethods
eTable 1. Complete Case Analysis
eTable 2. Association Between Sex-Specific Risk Factors With the Risk of New-Onset Atrial Fibrillation Among Participants Free of Cardiovascular Disease at Baseline
eTable 3. Association Between Various Sex-Specific Risk Factors With the Risk of New-Onset Atrial Fibrillation Stratified by Body Mass Index
eTable 4. Association Between Sex-Specific Risk Factors With the Risk of New-Onset Atrial Fibrillation Additionally Adjusted for Sex-Hormones
eTable 5. Association Between Sex-Specific Risk Factors With the Risk of New-Onset Atrial Fibrillation Stratified by Baseline Age Categories
eTable 6. Sensitivity Analysis: Association Between Categorical Reproductive Span and New-Onset AF After Further Adjustment for Menopausal Age
eTable 7. Sensitivity Analysis: Association Between Categorical Number of Live Births and New-Onset AF

This supplemental material has been provided by the authors to give readers additional information about their work.

© 2022 Lu Z et al. *JAMA Network Open.*

eMethods

In brief, age, sex, ethnicity (recoded to 5 groups: White, Asian, Black, mixed, and other), education (recoded to 2 groups: university/college, and other), alcohol intake frequency (recoded to 3 groups: never, 1~6 times per week, and every day), smoking status (never, former, and current), and medication use of blood pressure lowing, cholesterol lowing, oral contraceptive and hormone replacement therapy were obtained from the touchscreen questionnaires. Blood pressure was automatically measured twice during an interview using an Omron 705 IT electronic blood pressure monitor. Height (in meters) and weight (in kilogram) were measured in the assessment centers, and body mass index (BMI) was calculated as weight in kilograms divided by weight in meters squared. Prevalent cardiometabolic disorders at baseline, including coronary heart disease [CHD; ICD-10 codes: I20-22,24,25], AF (ICD-10 codes: I48), stroke (ICD codes: I60-64), heart failure [HF; ICD-10 codes: I50] and diabetes mellitus [(DM) ICD-10 codes: E10-14] was defined by ICD-10 codes on basis of the hospital admission and/or primary care. All self-reported cases of prevalent cardiometabolic disorders at baseline were set to missing.

© 2022 Lu Z et al. *JAMA Network Open.*

**eTable 1.** Complete Case Analysis		
	Hazard ratio (95% confidence interval)	*P*-values
Age at menarche *(n=219,415)	1.00 (0.97-1.02)	.72
7 ~ 11 years old	1.05 (0.92-1.19)	.50
12 years old	Reference	-
13 ~ 18 years old	1.03 (0.92-1.15)	.60
Irregular menstrual cycle, yes/no (n=38,677)	1.39 (0.96-2.02)	.08
Menopause, yes/no (n=126,540)	1.20 (0.98-1.48)	.08
Age at menopause † (n=80,626)	**0.94 (0.90-0.99)**	.02
< 35 years old	**2.92 (1.75-4.86)**	<.0001
35 ~ 44 years old	**1.24 (1.06-1.44)**	<.001
45 ~ 49 years old	0.99 (0.88-1.11)	.83
50 ~ 54 years old	Reference	-
55 ~ 59 years old	0.98 (0.86-1.11)	.76
≥ 60 years old	**1.49 (1.04-2.14)**	.03
Years after menopause † (n=80,626)	**1.06 (1.01-1.11)**	.02
Age at first live birth † (n=95,253)	**0.82 (0.86-0.97)**	<.01
Stillbirth, yes/no (n=222,941)	1.10 (0.87-1.40)	.40
Number of live births (n=141,783)	1.00 (0.97-1.04)	.85
None	**1.15 (1.03-1.29)**	.01
1~2	Reference	-
3	1.06 (0.95-1.17)	.29
4 ~ 6	**1.17 (1.01-1.35)**	.04
≥ 7	**2.33 (1.34-4.03)**	<.01
Reproductive years †‡ (n=78,852)	0.95 (0.91-1.00)	.05
≤ 20 years	**2.43 (1.34-4.41)**	<.01
21 ~ 30 years	**1.22 (1.04-1.43)**	.01
31 ~ 40 years	Reference	-
41 ~ 50 years	1.02 (0.91-1.13)	.78

Model was adjusted for baseline age, ethnicity, education, body mass index categories, total cholesterol, high density lipoprotein cholesterol, systolic blood pressure, diastolic blood pressure, smoking status, history of diabetes mellitus, history of coronary heart disease, history of heart failure, history of stroke, use of blood pressure lowering medication, use of cholesterol lowing medication, use of hormone replacement therapy (if applicable), and use of contraceptive medication (if applicable).

* Hazard ratios represent one unit increase in age at menarche with the risk of new-onset atrial fibrillation.

† Hazard ratios represent five unit increase in age at menopause, years after menopause, age at first live birth, reproductive years with the risk of new-onset atrial fibrillation.

‡ Reproductive years was defined as the difference between menopausal age and menarcheal age.

© 2022 Lu Z et al. *JAMA Network Open.*

eTable 2. Association Between Sex-Specific Risk Factors With the Risk of New-Onset Atrial Fibrillation Among Participants Free of Cardiovascular Disease at Baseline

	Hazard ratio (95% confidence interval)	*P*-values
Age at menarche * (n=219,415)	0.99 (0.97-1.01)	.45
Irregular menstrual cycle, yes/no (n=58,230)	1.36 (1.01-1.83)	.04
Menopause, yes/no (n=200,043)	1.14 (0.98-1.33)	.09
Age at menopause † (n=128,726)	0.94 (0.90-0.97)	<.001
Years after menopause † (n=128,726)	1.07 (1.03-1.11)	<.001
Age at first live birth † (n=150,894)	0.91 (0.87-0.96)	<.001
Stillbirth, yes/no (n=222,941)	1.08 (0.92-1.29)	.34
Number of live births (n=226,384)	0.99 (0.97-1.02)	.85
Reproductive years †‡ (n=125,859)	0.95 (0.91-0.98)	<.01

Model was adjusted for baseline age, ethnicity, education, body mass index categories, total cholesterol, high density lipoprotein cholesterol, systolic blood pressure, diastolic blood pressure, smoking status, history of diabetes mellitus, history of coronary heart disease, history of heart failure, history of stroke, use of blood pressure lowering medication, use of cholesterol lowing medication, use of hormone replacement therapy (if applicable), and use of contraceptive medication (if applicable).

* Hazard ratios represent one unit increase in age at menarche with the risk of new-onset atrial fibrillation.

† Hazard ratios represent five unit increase in age at menopause, years after menopause, age at first live birth, reproductive years with the risk of new-onset atrial fibrillation.

‡ Reproductive years was defined as the difference between menopausal age and menarcheal age.

**eTable 3.** Association Between Various Sex-Specific Risk Factors With the Risk of New-Onset Atrial Fibrillation Stratified by Body Mass Index					
	BMI categories				*P*-value interaction
	BMI<18.5	18.5≤BMI<25	25≤BMI<30	BMI≥30	
Age at menarche *	0.93 (0.74-1.18)	0.98 (0.95-1.02)	1.02 (0.98-1.05)	1.00 (0.97-1.03)	.90
Irregular menstrual cycle	-	0.97 (0.55-1.71)	1.62 (0.94-2.79)	1.45 (0.94-2.23)	.51
Menopause	1.49 (0.15-14.6)	1.39 (1.05-1.84)	1.15 (0.88-1.49)	0.96 (0.76-1.22)	<.0001
Age at menopause †	0.87 (0.57-1.33)	0.92 (0.86-0.99)	0.96 (0.90-1.02)	0.97 (0.91-1.03)	.19
Years after menopause †	1.15 (0.75-1.75)	1.09 (1.02-1.17)	1.04 (0.98-1.11)	1.03 (0.97-1.09)	.13
Age at first live birth †	0.77 (0.40-1.46)	0.95 (0.87-1.03)	0.89 (0.83-0.96)	0.96 (0.89-1.03)	.60
Stillbirth, yes/no	0.90 (0.59-1.37)	0.99 (0.70-1.39)	1.06 (0.82-1.39)	1.12 (0.87-1.43)	.90
Number of live births	1.06 (0.76-1.46)	0.97 (0.92-1.02)	1.02 (0.98-1.07)	*1.03 (0.99-1.07)*	.21
Reproductive years †‡	0.92 (0.61-1.39)	0.95 (0.88-1.01)	0.96 (0.91-1.01)	0.98 (0.93-1.04)	.24

Model was adjusted for baseline age, ethnicity, education, body mass index, total cholesterol, high density lipoprotein cholesterol, systolic blood pressure, diastolic blood pressure, smoking status, history of diabetes mellitus, history of coronary heart disease, history of heart failure, history of stroke, use of blood pressure lowering medication, use of cholesterol lowing medication, use of hormone replacement therapy (if applicable), and use of contraceptive medication (if applicable).

* Hazard ratios represent one unit increase in age at menarche with the risk of new-onset atrial fibrillation.

† Hazard ratios represent five unit increase in age at menopause, years after menopause, age at first live birth, reproductive years with the risk of new-onset atrial fibrillation.

‡ Reproductive years was defined as the difference between menopausal age and menarcheal age.

© 2022 Lu Z et al. *JAMA Network Open.*

eTable 4. Association Between Sex-Specific Risk Factors With the Risk of New-Onset Atrial Fibrillation Additionally Adjusted for Sex-Hormones

	Multivariate adjusted model
	Hazard ratio (95% confidence interval)
Age at menarche *	1.01 (0.98-1.03)
Irregular menstrual cycle, yes/no	1.41 (1.02-1.96)
Menopause, yes/no	1.14 (0.96-1.36)
Age at menopause †	0.96 (0.92-1.00)
Years after menopause †	1.04 (1.00-1.09)
Age at first live birth †	0.92 (0.87-0.97)
Stillbirth, yes/no	1.07 (0.91-1.26)
Number of live births	1.02 (0.99-1.05)
None	**1.11 (1.02-1.20)**
1~2	Reference
3	**1.09 (1.01-1.17)**
4 ~ 6	**1.16 (1.04-1.29)**
≥ 7	**1.64 (1.03-2.62)**
Reproductive years †‡	0.96 (0.92-1.00)

Model was adjusted for baseline age, ethnicity, education, body mass index categories, total cholesterol, high density lipoprotein cholesterol, systolic blood pressure, diastolic blood pressure, smoking status, history of diabetes mellitus, history of coronary heart disease, history of heart failure, history of stroke, use of blood pressure lowering medication, use of cholesterol lowing medication, use of hormone replacement therapy (if applicable), use of contraceptive medication (if applicable), and blood levels of testosterone and SHBG.

* Hazard ratios represent one unit increase in age at menarche with the risk of new-onset atrial fibrillation.

† Hazard ratios represent five unit increase in age at menopause, years after menopause, age at first live birth, reproductive years with the risk of new-onset atrial fibrillation.

‡ Reproductive years was defined as the difference between menopausal age and menarcheal age.

© 2022 Lu Z et al. *JAMA Network Open.*

**eTable 5.** Association Between Sex-Specific Risk Factors With the Risk of New-Onset Atrial Fibrillation Stratified by Baseline Age Categories							
	Age categories at baseline						*P*-value interaction
	≤ 45 years old	46-50 years old	51-55 years old	56-60 years old	61-65 years old	> 65 years old	
Age at menarche * (N=227,319)	1.04 (0.92-1.18)	0.97 (0.88-1.06)	1.01(0.95-1.07)	0.99 (0.94-1.03)	1.01(0.97-1.04)	1.00 (0.97-1.03)	.31
Irregular menstrual cycle, yes/no (N=58,843)	0.76 (0.36-1.61)	1.30 (0.83-2.03)	2.37 (1.40-4.02)	-	-	-	.03
Menopause, yes/no (N=206,886)	2.95 (1.26-6.89)	1.18 (0.79-1.77)	1.35 (1.00-1.82)	1.21 (0.62-2.34)	0.79 (0.47-1.31)	0.96 (0.64-1.45)	.09
Age at menopause † (N=134,419)	-	0.66 (0.47-0.92)	0.88 (0.75-1.03)	0.88 (0.81-0.97)	1.00 (0.94-1.06)	0.96 (0.90-1.01)	.08
Years after menopause † (N=134,419)	-	1.51 (1.08-2.11)	1.14 (0.98-1.34)	1.13 (1.04-1.23)	1.00 (0.94-1.06)	1.05 (0.99-1.11)	.18
Age at first live birth † (N=156,773)	0.88 (0.65-1.19)	0.91 (0.75-1.11)	0.81 (0.71-0.93)	0.92 (0.83-1.02)	0.99 (0.92-1.06)	0.89 (0.82-0.96)	.05
Number of live births (N=234,531)	0.93 (0.78-1.10)	1.1 (0.98-1.23)	1.02 (0.94-1.10)	0.98 (0.92-1.04)	0.98 (0.94-1.03)	1.04 (1.00-1.08)	.91
Reproductive years † ‡ (N=131,449)	-	0.70 (0.50-0.99)	0.91 (0.78-1.06)	0.91 (0.83-0.99)	1.00 (0.95-1.06)	0.96 (0.91-1.02)	.21

Model was adjusted for age, ethnicity, education, body mass index, total cholesterol, high density lipoprotein cholesterol, systolic blood pressure, diastolic blood pressure, smoking status, history of diabetes mellitus, history of coronary heart disease, history of heart failure, history of stroke, use of blood pressure lowering medication, use of cholesterol lowing medication, use of hormone replacement therapy (if applicable), and use of contraceptive medication (if applicable).

* Hazard ratios represent one unit increase in age at menarche with the risk of new-onset atrial fibrillation.

† Hazard ratios represent five unit increase in age at menopause, years after menopause, age at first live birth, reproductive years with the risk of new-onset atrial fibrillation.

‡ Reproductive years was defined as the difference between menopausal age and menarcheal age.

© 2022 Lu Z et al. *JAMA Network Open.*

eTable 6. Sensitivity Analysis: Association Between Categorical Reproductive Span and New-Onset AF After Further Adjustment for Menopausal Age

	Hazard ratio (95% confidence interval)	*P*-values
Reproductive years (n=149,462)		
≤ 20 years	1.62 (0.92-2.82)	.09
21 ~ 30 years	**1.19 (1.01-1.40)**	.04
31 ~ 40 years	Reference	-
41 ~ 50 years	1.06 (0.95-1.18)	.28

Model was adjusted for baseline age, ethnicity, education, body mass index categories, total cholesterol, high density lipoprotein cholesterol, systolic blood pressure, diastolic blood pressure, smoking status, history of diabetes mellitus, history of coronary heart disease, history of heart failure, history of stroke, use of blood pressure lowering medication, use of cholesterol lowing medication, **and menopausal age.**

*Hazard ratios represent five unit increase in reproductive years with the risk of new-onset atrial fibrillation.

© 2022 Lu Z et al. *JAMA Network Open.*

**eTable 7.** Sensitivity Analysis: Association Between Categorical Number of Live Births and New-Onset AF		
	Model 1	Model 2
Number of live births		
None	**1.15 (1.06-1.27)**	**1.09 (1.00-1.18)**
1~2	Reference	Reference
3	**1.14 (1.04-1.25)**	**1.08 (1.00-1.16)**
4 ~ 6	**1.16 (1.01-1.34)**	**1.13 (1.01-1.26)**
≥ 7	0.91 (0.38-2.18)	1.56 (0.98-2.49)

Model 1 was derived from **a sub-sample excluding women with ever stillbirth, spontaneous miscarriage, or termination**. Model was adjusted for baseline age, ethnicity, education, body mass index categories, total cholesterol, high density lipoprotein cholesterol, systolic blood pressure, diastolic blood pressure, smoking status, history of diabetes mellitus, history of coronary heart disease, history of heart failure, history of stroke, use of blood pressure lowering medication, and use of cholesterol lowing medication.

Model 2 was additionally adjusted for baseline age, ethnicity, education, body mass index categories, total cholesterol, high density lipoprotein cholesterol, systolic blood pressure, diastolic blood pressure, smoking status, history of diabetes mellitus, history of coronary heart disease, history of heart failure, history of stroke, use of blood pressure lowering medication, use of cholesterol lowing medication, **and Townsend index.**

© 2022 Lu Z et al. *JAMA Network Open.*
